# Assessing NODM Patients for Early PDAC Diagnosis: Incidence of NODM Before PDAC Diagnosis and Subsequent PDAC Risk

**DOI:** 10.1002/cam4.70878

**Published:** 2025-05-12

**Authors:** Satish Munigala, Benjamin Bowe, Divya S. Subramaniam, Hong Xian, Arna N. Gowda, Sunil G. Sheth, Rajiv Chhabra, Thomas E. Burroughs, Banke Agarwal

**Affiliations:** ^1^ College for Public Health and Social Justice Saint Louis University Saint Louis Missouri USA; ^2^ Department of Internal Medicine Washington University in St. Louis Saint Louis Missouri USA; ^3^ Clinical Epidemiology Center, Research and Education Service, VA Saint Louis, Health Care System Saint Louis Missouri USA; ^4^ Department of Health and Clinical Outcomes Research Saint Louis University School of Medicine Saint Louis Missouri USA; ^5^ Advanced HEAlth Data (AHEAD) Institute Saint Louis University Saint Louis Missouri USA; ^6^ Department of Epidemiology & Biostatistics, College for Public Health and Social Justice Saint Louis University Saint Louis Missouri USA; ^7^ Georgetown University Washington DC USA; ^8^ Beth Israel Deaconess Medical Center, and Harvard Medical School Boston Massachusetts USA; ^9^ Saint Luke's Hospital and University of Missouri Kansas City Kansas City Missouri USA; ^10^ Department of Gastroenterology SSM St. Anthony's Hospital Oklahoma City Oklahoma USA

**Keywords:** early diagnosis, high‐risk group, new‐onset diabetes, pancreatic cancer risk

## Abstract

**Background:**

New‐Onset Diabetes Mellitus (NODM) is often an early manifestation of pancreatic cancer (Pancreatic Ductal Adenocarcinoma, PDAC). However, there is limited information about (1) the duration prior to PDAC diagnosis when the annual incidence of NODM starts significantly exceeding that in age‐matched controls, (2) the percentage of PDAC patients diagnosed with NODM in the years preceding, and (3) the risk of PDAC following NODM in time when the PDAC risk is significantly higher than in controls.

**Methods:**

Using the nationwide VA database, we evaluated the annual incidence of NODM for 15 years preceding the PDAC diagnosis and in the age‐ and sex‐matched controls (1:5 matching). In the second part, we evaluated the long‐term risk and predictors of PDAC in NODM patients and controls.

**Results:**

The case–control study comprised 8198 PDAC patients and 40,992 matched controls. The higher annual incidence of NODM in PDAC patients was statistically significant up to 15 years before PDAC diagnosis. 69.2% of PDAC patients had NODM in the preceding 15 years versus 38.0% of controls. PDAC risk in the 15 years following NODM was 0.60% compared to 0.13% in the controls (aHR 3.83, 95% CI 3.68–3.98, *p* < 0.001). The risk of PDAC is more pronounced in the 1 year following NODM (aHR 9.07, 95% CI 8.33–9.87) than the subsequent 5 years (aHR 2.98, 95% CI 2.82–3.15).

**Conclusion:**

NODM pre‐dates PDAC diagnosis in most patients with PDAC. Further evaluation of NODM patients for PDAC has the potential to become a feasible strategy for diagnosing more early‐stage resectable PDACs.

## Graphical abstract

Evaluation of New‐Onset Diabetes Mellitus (NODM) for Pancreatic Ductal Adenocarcinoma at the onset of NODM and subsequent surveillance for 3–12 years would be a potential strategy for early diagnosis and improved outcomes in patients with pancreatic cancer.

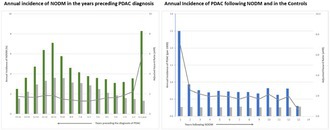

## 1 Introduction

Pancreatic Ductal Adenocarcinoma (PDAC) outcomes have shown limited progress over the last 5 decades [1, 2, 3]. Early diagnosis to detect surgically resectable tumors has proven to be an unsurmountable challenge, with < 10% of PDACs being resectable at the time of diagnosis. Furthermore, most resectable PDACs are asymptomatic [4, 5]. Population‐based screening programs are limited due to the relatively low incidence of PDAC. Identifying higher‐risk population groups for screening and surveillance is therefore vital. This priority stems from the urgent need to diagnose PDAC early and improve overall outcomes [4].

New‐Onset Diabetes Mellitus (NODM) is often an early manifestation of PDAC and has recently evolved as a high‐risk group of significant interest. However, the reported PDAC rates following NODM exhibit considerable variability, ranging from 0.1% to 1.02% [6, 7, 8]. However, the Duration of Excess Risk (DER), which signifies the number of years before PDAC diagnosis that the incidence of NODM significantly exceeds that in age‐ and sex‐matched controls, lacks clarity. Additionally, the proportion of PDAC patients diagnosed with NODM during the DER, and who are eligible for targeted surveillance to enable early detection while the tumor is still surgically resectable, remains uncertain. There is also a lack of data on the time distribution of PDAC diagnoses following NODM, which is crucial for determining the ideal duration for surveillance.

Using the nationwide Veteran's Administration (VA) database, this study evaluated (a) the time duration before PDAC diagnosis when NODM incidence starts significantly exceeding the age and sex‐matched controls (DER, b) the percentage of PDAC patients diagnosed with NODM in the DER, (c) the incidence of PDAC following NODM in the time period equal to DER, and (d) patient factors that influence the likelihood of PDAC diagnosis in the NODM cohort.

## 2 Methods

### 2.1 Data Source

The Veterans Affairs Medical System (VA) inpatient and outpatient medical SAS datasets, including utilization data related to all encounters within the VA system from September 1999 through December 2015, were utilized. These datasets were used to ascertain detailed cohort participants' demographic characteristics, PDAC, and other comorbidity information based on the International Classification of Diseases, 9th Revision Clinical Modification (ICD‐9‐CM) diagnostic and procedure codes associated with inpatient and outpatient encounters.

### 2.2 Pancreatic Cancer

Pancreatic cancer was defined based on primary or secondary diagnosis codes (≥ 1 code) for adenocarcinoma of the pancreas (inpatient or outpatient, ICD 9 codes 157.0–157.4, 157.8, and 157.9). The date of the first ICD 9 diagnosis code was used as the date of the PDAC diagnosis.

### 2.3 New‐Onset Diabetes Mellitus

NODM (the first instance of diagnosis of Diabetes Mellitus) was defined using glycemic parameters meeting the following criteria: age ≥ 40, date of meeting criteria for diabetes on enrollment, and glycemic parameter(s) measured in the 3–18 months before screening (demonstrating absence of parameters for diabetes mellitus [PDM]) (Table S1). Patients < 40 years of age were excluded from the retrospective cohort as we had earlier reported that the risk of PDAC was extremely low in that patient subset [9]. In this manuscript, we have used the term NODM to refer to the date of initial diagnosis of DM (even when followed over 10 years or more) and differently from other authors who have used NODM to allude to recent onset DM.

### 2.4 Covariates

Acute pancreatitis (AP) is defined based on the inpatient diagnoses' codes for AP (primary or secondary, ICD 9 code 577.0); chronic pancreatitis (CP) is defined based on primary or secondary diagnoses codes (inpatient or outpatient, ICD 9 codes 577.1), presence of gallstones (ICD codes 574, 574.1, 574.3, 574.5, 574.7, 574.8, 574.9), history of heavy alcohol consumption (ICD 9 codes 303.0 [alcohol dependence], 303.9 [alcohol addiction], 305.0 [acute alcohol intoxication]), smoking (based on personal history of smoking: current/past/non‐smoker) and demographic variables (age at the time of entry into the study, sex: male/female, race: white/black/other) were also evaluated as these are known risk factors for PDAC.

### 2.5 Statistical Analysis

For Study Part I, we used a case–control design to evaluate the annual incidence of NODM (new diagnosis of DM) prior to PDAC diagnosis. For Study Part II, we evaluated the annual incidence of PDAC diagnosis in the years following NODM. The Data S1 describes detailed methodology, including exclusion criteria, cohort selection, and statistical analysis for Study Parts I and II.

All analyses used SAS version 9.3 (SAS Inc., Cary, NC). Significance tests were performed using a 2‐tailed hypothesis, and the level of significance (α) was set to 0.05.

## 3 Results

### 3.1 Part 1: Case–Control Study

#### 3.1.1 Patient Characteristics

**FIGURE 1:**
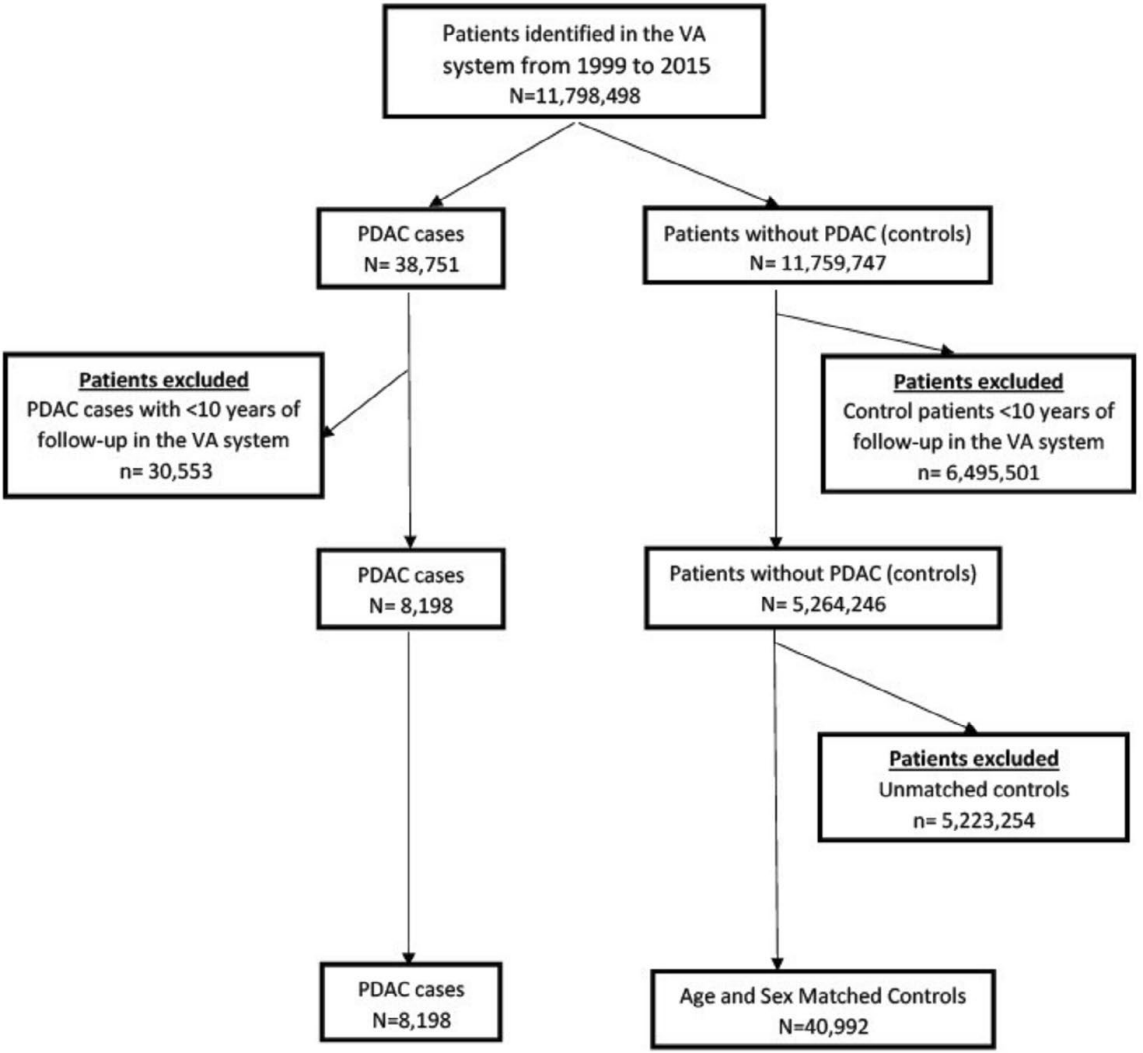
Selection of participants for Study I: case–control study comparing the annual incidence of NODM in the years preceding the diagnosis of PDAC. NODM, New‐Onset Diabetes Mellitus; PDAC, Pancreatic Ductal Adenocarcinoma.

This study included 49,190 veterans comprising 8198 patients with PDAC and 40,992 age‐ and sex‐matched controls (Figure 1). Their median age was 70, and 96.8% were males (Table 1). In the PDAC group, 69% of patients (5673 of 8198) had NODM diagnosed within 15 years preceding PDAC diagnosis compared to 38.0% of the controls (*n* = 15,576 of 40,992, *p* < 0.001).

**TABLE 1:** Patient characteristics of pancreatic cancer cases and matched controls.

	PDAC cases	Age and sex matched controls	*p*	All study patients^a^
*N* (%)	8198 (100)	40,992 (100)		49,190
Age in years (Median, interquartile range)	70 (64–80)	70 (64–80)	0.976	70 (64–80)
Sex				
Male	7935 (96.8)	39,676 (96.8)	0.992	47,611 (96.8)
Female	263 (3.2)	1316 (3.2)		1579 (3.2)
Race				
White	6129 (75.3)	32,466 (80.9)	Reference	38,595 (80.0)
Black	1557 (19.1)	5678 (14.2)	< 0.001	7235 (15.0)
Other	458 (5.6)	1970 (4.9)	< 0.001	2428 (5.0)
Smoking				
Current	3443 (42.2)	12,777 (34.2)	< 0.001	16,220 (35.6)
Past	1661 (20.4)	9080 (24.3)	0.031	10,741 (23.6)
Alcohol	2167 (26.4)	6829 (16.7)	< 0.001	8996 (18.3)
Gallstone disease	1273 (15.5)	1763 (4.3)	< 0.001	3036 (6.2)
NODM	5673 (69.2)	15,576 (38.0)	< 0.001	21,249 (43.2)

*Note:* Age‐age at the time of PDAC diagnosis or index date in controls.

Abbreviations: NODM, New‐Onset Diabetes Mellitus; PDAC, Pancreatic Ductal Adenocarcinoma.

^a^ Race missing in ~1.9% (*n* = 932) of all patients included in the study.

**FIGURE 2:**
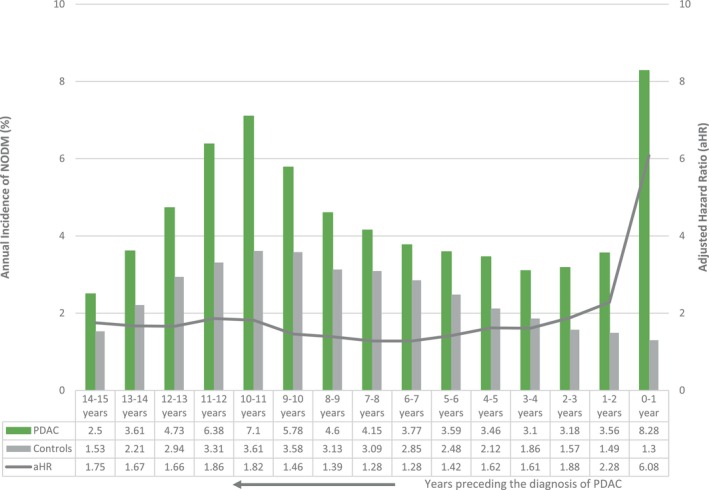
Annual incidence of NODM in the years preceding a PDAC diagnosis.

As seen in Figure 2 (and Table S2), the annual incidence of NODM remained significantly higher in the PDAC group than in the controls for up to 15 years before the PDAC diagnosis. The incidence of NODM in the PDAC group had a bimodal distribution, with a peak in the year preceding the PDAC diagnosis and another peak about 10 years earlier. As seen in Table S2, for the Years 7–15, the adjusted HR for NODM ranged from 1.28 to 1.86. From Years 6 to 2 before PDAC diagnosis, the adjusted HR for NODM increases from 1.28 to 2.28 and then increases dramatically in the year prior (aHR 6.08, 5.41–6.83) to PDAC diagnosis. This occurs despite a continued decrease in NODM incidence in the age‐matched controls during the same time.

Table 2 summarizes the observed, expected, and excess number of NODM cases in the 7 years leading up to PDAC diagnosis. The difference in annual incidence of NODM amongst patients with PDAC and age‐sex‐matched controls reached a low at Year 7. If the difference were entirely due to long‐standing DM promoting PDAC, then sometime between Years 7 to 1 prior to PDAC diagnosis, the risk of NODM would become identical in the two groups. This increase from Years 7 to 1 before PDAC diagnosis is likely because PDAC somehow leads to the development of DM (Type 3c DM). As seen in Table 2, the proportion of excess cases of NODM (most likely Type 3c DM) increases steadily from Years 7 to 1 before PDAC diagnosis. 84.3% or more of NODM within the year of PDAC diagnosis are directly related to PDAC.

**TABLE 2:** Estimated number of cases of Type 3c Diabetes Mellitus in the 7 years leading up to pancreatic adenocarcinoma diagnosis in the study patients.

	Patients at risk	Observed (*n*)	Expected (*n*)	Excess^a^ (*n*)	Excess (%)
Year 1	8198	679	107	572	84.3%
Year 2	7519	292	114	178	61.1%
Year 3	7227	261	117	144	55.1%
Year 4	6966	254	135	119	46.8%
Year 5	6712	284	152	132	46.6%
Year 6	6428	294	174	120	40.7%
Year 7	6134	309	198	111	35.9%

^a^ Excess number of PDAC cases amongst NODM patients was calculated using the percentage of PDAC cases diagnosed in the age‐sex‐matched controls for each year, respectively, from Table S2.

### 3.2 Part II: PDAC Risk Following NODM

#### 3.2.1 Patient Characteristics

**FIGURE 3:**
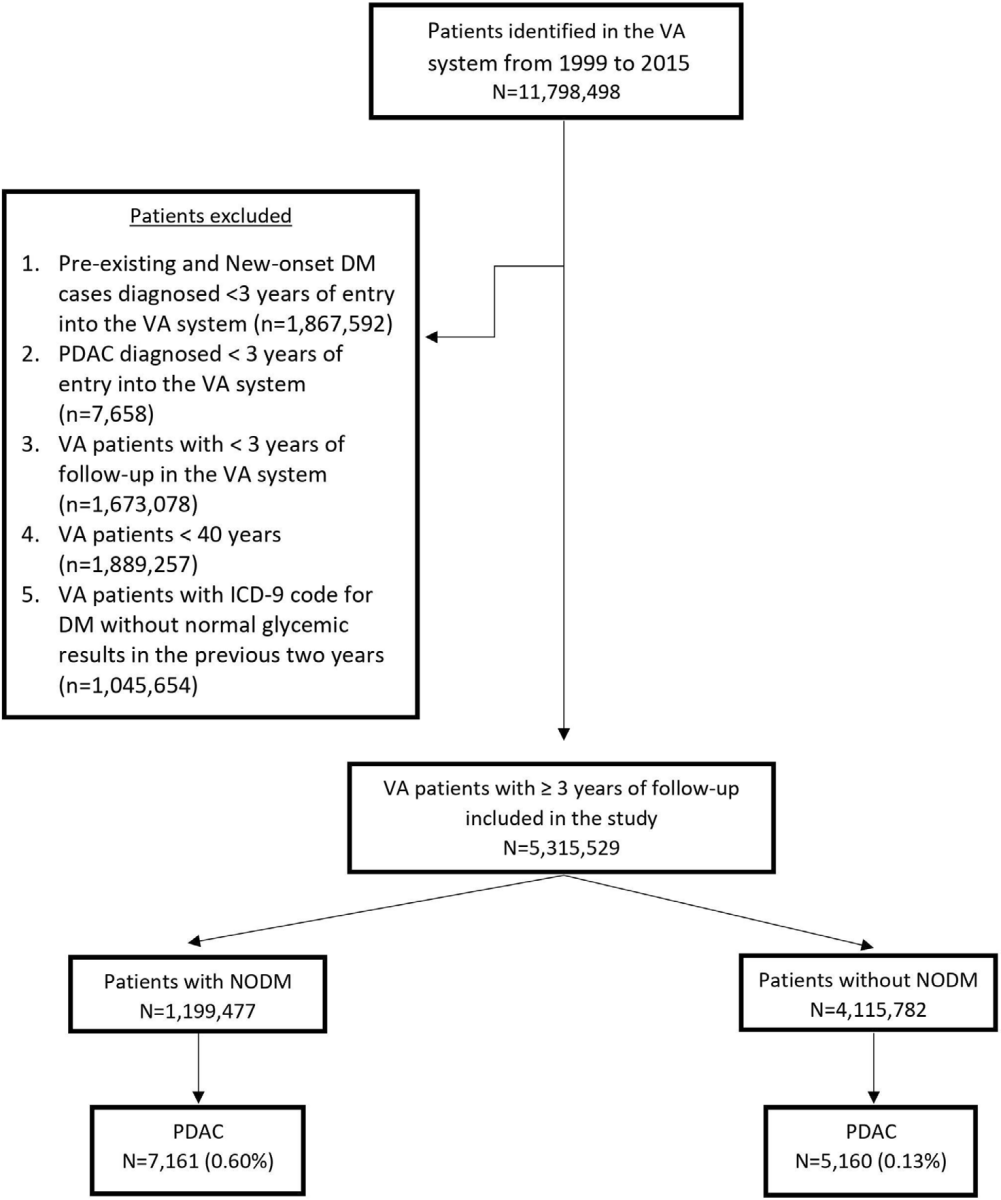
Selection of cohort for Study II: determination of risk of PDAC following NODM.

As shown in Figure 3, this cohort comprised 5,315,259 veterans, 1,199,477 with NODM and the remaining 4,115,782 veterans (controls) followed for up to 15 years. Median follow‐up was 8.7 years and 10 years, respectively, for the NODM and the control groups. Patient characteristics are summarized in Table S3. PDAC was diagnosed in 5160 veterans (0.13%) in the control group and 7161 veterans in the NODM group (0.6%).

#### 3.2.2 PDAC Incidence by NODM Status

**FIGURE 4:**
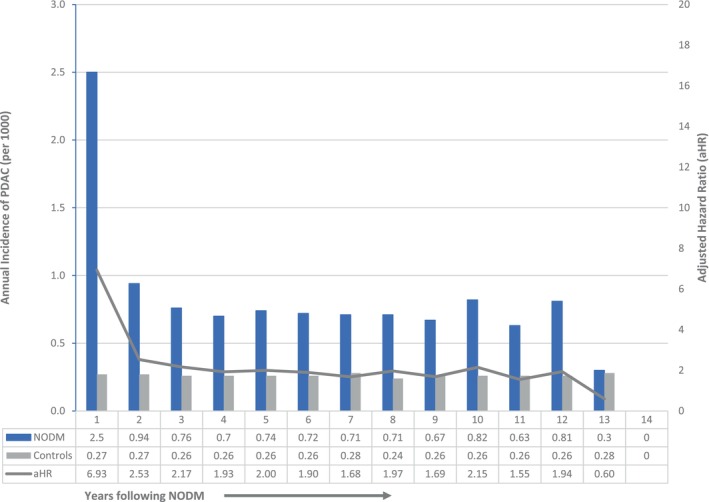
Annual Incidence of PDAC following NODM and in controls.

The annual incidence of PDAC for each year following NODM is summarized in Figure 4 (and Table S4). The incidence of PDAC was highest in the first year (adjusted HR 9.07, 95% CI 8.33–9.87), came down dramatically in the second year (adjusted HR 3.40, 95% CI 3.06–3.77), and stayed elevated but stable in years 3–12, and became comparable to controls in the 13th year. The number of patients at risk after 12 years was relatively small, and therefore, the abrogation of the increased PDAC risk in Years 13 and 14 is not definitive.

**FIGURE 5:**
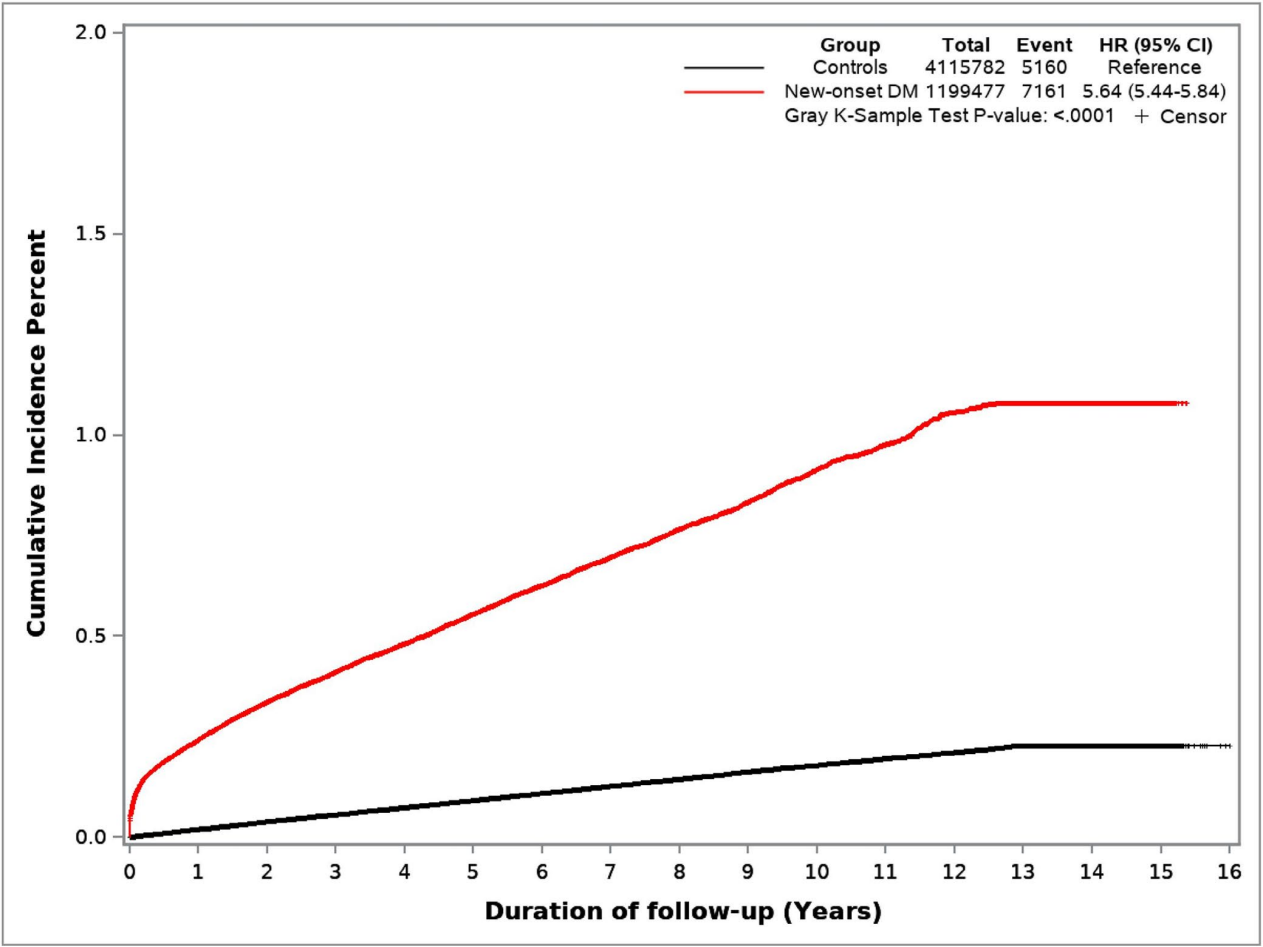
Cumulative Incidence of PDAC following NODM. Hazard ratios presented on the figures are unadjusted hazard ratios from the Cox‐proportional model.

**FIGURE 6:**
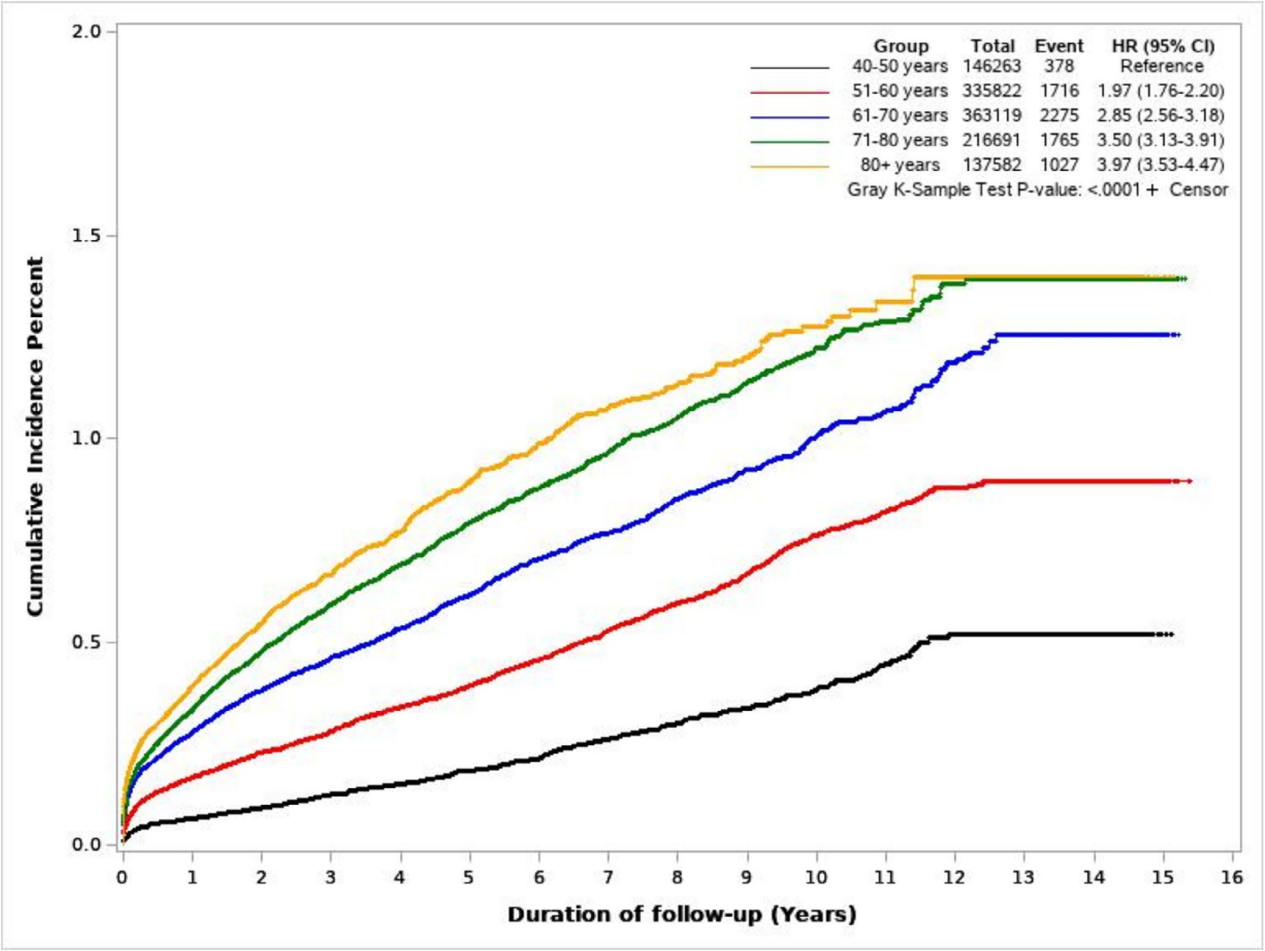
Cumulative incidence of PDAC following NODM based on the decade of life the patient was in at the time of NODM diagnosis.

Figure 5 shows cumulative incidence curves of PDAC for NODM and control study groups. Figure 6 illustrates the cumulative incidence of PDAC following NODM based on the decade of life the patient was in at the time of NODM diagnosis. PDAC risk following NODM increased progressively from the fifth decade of life (HR 1.50, 1.36–1.66) at the time of diagnosis to the ninth decade (HR 6.39, 5.99–6.81).

In total, 7161 patients with NODM were diagnosed with PDAC during the study follow‐up and comprised 58% of all PDACs (*n* = 12,321) diagnosed in the entire cohort during that period. Table 3 details the incidence of PDAC diagnosis within 1 and 2 years following NODM in patients in their fifth to ninth decades. 2814, 3762, and 4446 patients were diagnosed with PDAC within 1, 2, and 3 years following the diagnosis of NODM. These comprised 0.23%, 0.32%, and 0.39% of patients at risk and comprised 78%, 72%, and 69% of all new diagnoses of PDAC in the entire cohort in the corresponding time periods.

**TABLE 3:** Number of patients diagnosed with PDAC in the first 2 years following NODM.

Age group	Patients at risk	PDAC diagnosed in first year; *n* (%)	PDAC diagnosed in second year; *n* (%)	PDAC diagnosed in Years 1+ 2; *n* (%)
40–49	146,169	94 (0.06)	34 (0.03)	128 (0.09)
50–59	335,373	549 (0.16)	178 (0.06)	727 (0.22)
60–69	362,147	972 (0.27)	316 (0.10)	1288 (0.37)
70–79	215,996	695 (0.32)	259 (0.14)	954 (0.46)
80+	137,078	504 (0.36)	161 (0.12)	665 (0.48)
Total	1,196,663	2814 (0.23)	948 (0.09)	3762 (0.32)

Using a cause‐specific hazard model, and adjusting for age, sex, race, smoking status, alcohol history, and history of gallstones, NODM patients had a higher PDAC risk in the subsequent 12 years (adjusted HR 3.83, 95% CI 3.68–3.98, *p* < 0.001, NODM vs. controls). On multivariable analysis (Table S5), amongst NODM patients, male sex, increasing age, smoking, history of gallstone disease, AP, and CP were significantly associated with the subsequent risk of PDAC (*p* < 0.001).

## 4 Discussion

Using the case–control methodology, we found that in patients with PDAC, the annual incidence of NODM exceeded that in age‐ and sex‐matched controls up to 15 years preceding the PDAC diagnosis. NODM was diagnosed in 69.2% of PDAC patients compared to 38% in age‐ and sex‐matched controls during the study period. In a separate cohort, we noted that 0.60%, 0.23%, and 0.32% of patients with NODM were diagnosed with PDAC in the 12, 1, and 2 years, respectively, following NODM diagnosis. They comprised 58%, 78%, and 72% of all PDACs diagnosed in the entire cohort during the corresponding time periods. PDAC risk following NODM diagnosis increased markedly with each decade of life after the fifth decade. Gallstone disease, AP, and CP were significant and the strongest predictors of subsequent PDAC diagnosis in a multivariable analysis.

Early diagnosis of PDAC continues to be a considerable challenge. The size of PDAC at the time of diagnosis has barely improved in the past 50 years despite dramatic advances in medical imaging [1, 2, 3]. Pancreatic cancer tends to remain asymptomatic until it reaches an advanced stage, and therefore, individuals with early‐stage tumors rarely seek or come to medical attention. Population screening for PDAC is not feasible due to its relatively low prevalence rates [10, 11]. Therefore, by identifying clinical presentations that are associated with a higher risk of PDAC and evaluating patients with these presentations, there is a potential to enhance the early detection of PDAC and diagnose more early‐stage tumors. At present, the recognized high‐risk groups for PDAC, such as patients with mucinous pancreatic cysts and patients with family history or genetic predisposition for PDAC, account for only a tiny fraction of all PDAC cases [5]. We had earlier reported that routinely evaluating patients with AP and newly diagnosed CP for PDAC might be worth further study to aid in its early diagnosis [9, 12].

NODM has lately gained significant attention as a high‐risk group and a potential target for PDAC surveillance. Recognizing its importance, the National Cancer Institute (NCI) has identified exploring the relationship between diabetes mellitus (DM) and PDAC as one of the top priorities in PDAC research [10]. The reported PDAC risk following NODM ranges from 0.10% to 1.02% [7, 8, 13, 14, 15, 16, 17, 18, 19, 20, 21, 22, 23, 24]. Yet, there is no clarity on (1) how far back in time the incidence of NODM in patients diagnosed with PDAC becomes significantly higher than that of the matched controls (DER), and (2) how many PDAC cases have NODM in the DER to enable estimation of what proportion of PDACs could be diagnosed early based on this approach.

We noted a significantly higher incidence of NODM in PDAC patients compared to age‐ and sex‐matched controls up to 15 years before PDAC diagnosis. There are bimodal peaks of the annual incidence of NODM, one in the year preceding the PDAC diagnosis and the other 10 years prior. It has been established that patients with long‐standing diabetes mellitus (> 5 years) have a higher risk of pancreatic cancer (1.5–1.7‐fold) [25]. We similarly noticed a higher relative risk of PDAC following 7–12 years of NODM. Suppose the patients living with diabetes have a higher PDAC risk; In that case, it is expected that the ‘apparent’ incidence of NODM would be higher in years preceding PDAC diagnosis compared to age‐ and sex‐matched controls. As per our calculations, this higher PDAC risk following long‐standing DM is sufficient to explain our cohort's increased annual incidence of NODM 7–15 years before PDAC diagnosis.

The hazard ratio for NODM increases gradually from 1.28 at 6 years to 2.28 at 2 years before PDAC, followed by a dramatic increase to 6.06 in the year before PDAC diagnosis. This happened despite a gradual and progressive decrease in NODM incidence in the age‐sex‐matched controls during the same period. Based on the temporal profile, the excess number of NODMs up to 6 years before PDAC diagnosis is likely due to the pancreatic tumor (Type 3c DM, as summarized in Table 2). Unfortunately, there is still no biochemical test to diagnose Type 3c DM. This likely also represents the Duration of Excess Risk of NODM due to PDAC. 85% of NODM cases in the year preceding PDAC diagnosis had Type 3c DM. It seems difficult to believe that the excess risk for NODM can start up to 6 years before PDAC diagnosis. A case report documented a patient who developed NODM and deep vein thrombosis 6 years before PDAC diagnosis [26]. Radiologic findings of ‘focal pancreatic enhancement’ and ‘focal pancreatic atrophy’ have been reported to occur 3.3 and 4.6 years (mean duration) before the clinical diagnosis of PDAC [27, 28]. It could be that some pancreatic tumors grow very slowly (average 11.7 years) or because even very early‐stage pancreatic cancer cells can somehow cause DM [29]. The markedly higher risk of NODM in the year preceding PDAC diagnosis suggests that PDAC has a direct and potent role. The potential mechanisms and signaling pathways that underlie the relationship between adult‐onset DM and PDAC are under active investigation. Several molecules, including adrenomedullin, adipokines (including lipocalin, leptin, and adiponectin), and calprotectin, have been reported to be associated with PDAC‐associated diabetes mellitus [30]. Identifying molecular mechanisms and cellular pathways through which PDAC causes DM could uncover new avenues for early detection and intervention.

The 3‐year incidence of PDAC diagnosis following NODM has been a matter of considerable debate. It has been suggested that the 3‐year probability of PDAC following NODM is 1% or higher. We compiled a list of cohort studies that reported the 3‐year PDAC risk after NODM, or the manuscript had data that allowed this estimation (Table S6). As is apparent, the two studies that reported 0.85% and 1.02% risk were both from the same database (Mayo Clinic, Olmsted County) [7, 21], and had far fewer patients than the other studies, both from the US and overseas, which showed much lower 3‐year PDAC risk in NODM patients [13, 14, 16, 18, 22, 23, 24, 31, 32]. Pooled data from these studies had a 0.28% risk of PDAC in 3 years after PDAC diagnosis. This is in line with the 3‐year PDAC risk in the present study and 0.25% that we noted in our previous manuscript [19].

Another point of debate is whether including patients based on glycemic parameters for diagnosing NODM (rather than based on physician diagnoses or using ICD codes) leads to the selection of patient cohorts with a higher 3‐year risk of PDAC. We tested this hypothesis in our database (Table S7). Group 1 comprises physician‐diagnosed patients with ICD codes for DM in addition to abnormal glycemic parameters. Group II only met the glycemic criteria. The 3‐year risk of PDAC was 0.31% and 0.41% in groups I and II, respectively (*p* < 0.001). Patients with abnormal glycemic parameters, irrespective of whether they have a physician diagnosis (ICD code for DM), are now referred to as glycemic New‐Onset Diabetes Mellitus (gNOD). Focusing on patients with gNOD for screening/surveillance for PDAC also helps cast a wider net by adding 734,069 more patients to the 465,408 patients with a physician‐diagnosed NODM.

Despite significant progress in imaging and treatment techniques, little improvement in their outcomes has occurred over the past 50 years. Therefore, serious consideration of novel approaches is warranted. A substantial body of data demonstrates that patients diagnosed with early‐stage PDAC experience significantly improved outcomes [1, 2, 3]. Surveillance with Endoscopic Ultrasound (EUS) and Magnetic Resonance Index (MRI) in patients with hereditary predisposition to PDAC has recently been reported to be associated with dramatically better survival in several studies [33, 34]. However, hereditary PDACs comprise only 5%–10% of all PDACs. A strategy using NODM as a high‐risk group for surveillance can potentially cast a wider net.

The pre‐test probability of PDAC in patients with NODM seems rather low and insufficient for screening/surveillance for PDAC. Enrichment of the cohort for screening based on age at NODM onset, weight change, and change in blood glucose (Enriching New‐Onset Diabetes for Pancreatic Cancer (ENDPAC)) has been suggested [21, 31, 35, 36]. One‐time evaluation for PDAC after NODM (with MRI scan or EUS) or limited surveillance for 2 years are other options to maximize yield and limit costs. It could help in early diagnosis of up to 70% of all PDACs in the entire cohort in the corresponding first 3 years (with 0.41% pre‐test probability). If MRI is used for initial evaluation, then patients who are noted to have subtle abnormalities such as (a) focal dilation of the pancreatic duct with abrupt cutoff, (b) focal parenchymal atrophy or enhancement, (c) acute idiopathic distal pancreatitis, and (d) vascular encasement should be further evaluated with EUS [27, 37]. Of course, if a focal pancreatic lesion is noted on MRI, EUS‐FNA can provide tissue diagnosis of PDAC so that treatment can be initiated with confidence [38]. Limiting PDAC evaluation only to those who meet the clinical criteria for Type 3c DM could also potentially increase the pre‐test probability of the PDAC‐targeted patient subset.

The potential benefit of evaluating patients with NODM for PDAC is not trivial since < 10% of all PDACs are currently diagnosed when surgery could meaningfully improve the outcomes [39]. Upon re‐evaluation of CT scans, tumors are much smaller and can be identified up to 2–3 years before PDAC diagnosis [40]. EUS will be very likely to perform much better than CT scans, with higher sensitivity and provide tissue diagnosis [38, 41]. Ability to detect and diagnose up to 70% sporadic PDACs early (up to 3 years earlier) when they are still surgically resectable would surely be a significant advance in pancreatic cancer management.

This study has limitations due to its retrospective design and the use of administrative data using ICD 9 codes for PDAC and other covariates. Our cohort of US veterans included 94% males, limiting the generalizability. The gender imbalance observed in the study is mainly due to the US veteran population being overwhelmingly male overall, although the percentage of women in recent years has improved. Due to the nature of this large study cohort, validation of the ICD 9 codes used for all patients with PDAC was not possible. We reviewed the medical charts of a random 100 PDAC patients, and the accuracy of the ICD‐9 codes for PDAC was 85%. Due to an insufficient number of PDAC events, we could not assess relative risk beyond the 14th year. As with any administrative database, any DM diagnosis made before the study entry and any PDAC diagnosis after the study ended could not be captured. However, to limit the bias due to the nature of the database, we applied a washout period of 2 years without abnormal glycemic parameters and without the use of anti‐diabetic medications before including patients in the study to ensure that patients with pre‐existing diabetes diagnosed outside of VA were not inadvertently included in the study.

Our study has several notable strengths, including a large sample size, long duration of follow‐up, controlling for smoking, heavy alcohol use status, and history of gallstones. NODM in our cohort was used to define the course of DM from its onset and subsequently over the years (in contrast to long‐standing diabetes, whose exact duration is not specified). Due to this, our data provides a more precise relationship between DM from its initial diagnosis and PDAC. NODM patients were not included in the control group before their NODM diagnosis. We also analyzed the PDAC risk in NODM patients in the context of AP and CP and found them to be significant predictors of the subsequent diagnosis of PDAC. They can be used to select NODM patients for further evaluation and surveillance. Despite the limitations mentioned above, the risk of PDAC in this database is consistent with recently published studies using different datasets without limitations of a VA database, including male predominance [8, 13, 14, 19]. Our data is derived from one of the largest databases in the US and has a meaningful duration of follow‐up (~16 years) and adequate numbers of patients with NODM (> 1 million) and PDAC (> 16,000).

In conclusion, our study reveals that PDAC‐related Type 3c DM can precede PDAC diagnosis by up to 6 years and could be a potent tool for the early diagnosis of PDAC. The pre‐test probability of PDAC in patients with NODM is still rather low. Still, it can be increased by selecting patients based on additional clinical features so that further evaluation and even surveillance of patients with NODM for PDAC become feasible. Evaluating PDAC at the onset of NODM could help in the early diagnosis of up to 70% of all PDAC patients and potentially improve outcomes. Further studies are required to evaluate its feasibility and efficacy.

## Author Contributions

**Satish Munigala:** conceptualization (lead), data curation (lead), formal analysis (lead), methodology (equal), project administration (equal), writing – original draft (equal), writing – review and editing (equal). **Benjamin Bowe:** conceptualization (supporting), methodology (supporting), writing – review and editing (supporting). **Divya S. Subramaniam:** conceptualization (supporting), investigation (supporting), writing – original draft (supporting), writing – review and editing (supporting). **Hong Xian:** methodology (supporting), writing – original draft (supporting), writing – review and editing (supporting). **Arna N. Gowda:** writing – review and editing (supporting). **Sunil G. Sheth:** conceptualization (supporting), writing – original draft (supporting), writing – review and editing (supporting). **Rajiv Chhabra:** writing – original draft (supporting), writing – review and editing (supporting). **Thomas E. Burroughs:** conceptualization (supporting), investigation (supporting), writing – original draft (supporting), writing – review and editing (supporting). **Banke Agarwal:** conceptualization (equal), investigation (equal), methodology (equal), writing – original draft (equal), writing – review and editing (equal).

## Disclosure

Satish Munigala accepts full responsibility for the study and has access to the data.

## Ethics Statement

This study was approved by the Saint Louis Veterans Affairs Medical Center.

## Conflicts of Interest

The authors declare no conflicts of interest.

## Supporting information


Tables S1–S7:

Data S1:

## Data Availability

Due to the nature of the research, data supporting this study's findings are not publicly available.
