# Supplementary material for: Assessing NODM Patients for Early PDAC Diagnosis: Incidence of NODM Before PDAC Diagnosis and Subsequent PDAC Risk
Source: Cancer Med. 2025 May 12;14(9):e70878. doi: 10.1002/cam4.70878 (PMC12066942; doi:10.1002/cam4.70878)
Supplement: Supplementary file 1 — Tables S1–S7: [file CAM4-14-e70878-s001.docx]

**Supplementary Data**

**Table S1.** Criteria for diagnosing New-Onset Diabetes in present study

| **Parameters of Diabetes Mellitus (PDMs)** | - FBG (≥126 mg/dL) - Random blood glucose (≥200 mg/dL) - HbA1c (≥6.5%) |
| --- | --- |
| **Diagnosis of Diabetes Mellitus** | **Any (1) of the following:**   - Any (2) PDMs present on consecutive or simultaneous testing - ICD-9 diagnosis code for diabetes preceded by at least (1) PDM - On antidiabetic medications preceded by at least (1) PDM |
| **Date of meeting criteria for DM** | **Whichever of the following is earliest:**   - Date of first of (2) consecutive or simultaneous measured PDMs - Date of first PDM if had an ICD-9 diagnosis code for diabetes following this measurement - Date of first PDM if patient is started on anti-DM medications following this measurement |
| **New-onset DM** | **Meets all the following criteria:**   - Age ≥40 - Date of meeting criteria for DM on enrollment - Have had glycemic parameter(s) measured in the 3–18 months before screening (demonstrating absence of PDM) |

**Table S2.** Risk (hazard ratio) of prior diagnosis of diabetes by year and pancreatic cancer status

|  | **PDAC cases** | | **Age- and Sex- Matched Controls** | | **Incidence per 1000 patient years** | | **Risk** | | |
| --- | --- | --- | --- | --- | --- | --- | --- | --- | --- |
|  | Patients at risk (n) | Patients diagnosed with NODM | Patients at risk (n) | Patients diagnosed with NODM | PDAC | Controls | Adjusted Hazard Ratio | 95% Confidence Interval | P value |
| Year 1 | 8198 | 679 (8.28) | 40992 | 532 (1.30) | 88.12 | 13.06 | 6.08 | 5.41-6.83 | <0.001 |
| Year 2 | 7519 | 292 (3.88) | 40460 | 610 (1.51) | 39.62 | 15.19 | 2.28 | 1.97-2.63 | <0.001 |
| Year 3 | 7227 | 261 (3.61) | 39850 | 644 (1.62) | 36.79 | 16.29 | 1.88 | 1.62-2.19 | <0.001 |
| Year 4 | 6966 | 254 (3.65) | 39204 | 762 (1.94) | 37.14 | 19.62 | 1.61 | 1.39-1.87 | <0.001 |
| Year 5 | 6712 | 284 (4.23) | 38424 | 867 (2.26) | 43.21 | 22.85 | 1.62 | 1.41-1.86 | <0.001 |
| Year 6 | 6428 | 294 (4.57) | 37422 | 1015 (2.71) | 46.81 | 27.62 | 1.42 | 1.24-1.63 | <0.001 |
| Year 7 | 6134 | 309 (5.04) | 36032 | 1165 (3.23) | 51.69 | 31.31 | 1.28 | 1.13-1.46 | <0.001 |
| Year 8 | 5825 | 340 (5.84) | 34137 | 1265 (3.71) | 60.06 | 38.38 | 1.28 | 1.13-1.45 | <0.001 |
| Year 9 | 5485 | 377 (6.87) | 31650 | 1282 (4.05) | 70.98 | 42.55 | 1.39 | 1.23-1.56 | <0.001 |
| Year 10 | 5108 | 474 (9.28) | 28441 | 1467 (5.16) | 97.27 | 55.44 | 1.46 | 1.31-1.63 | <0.001 |
| Year 11 | 4634 | 582 (12.56) | 24360 | 1478 (6.07) | 143.73 | 68.03 | 1.82 | 1.65-2.01 | <0.001 |
| Year 12 | 3463 | 523 (15.10) | 19126 | 1358 (7.10) | 179.85 | 82.01 | 1.86 | 1.67-2.06 | <0.001 |
| Year 13 | 2370 | 388 (16.37) | 14034 | 1204 (8.58) | 202.97 | 103.80 | 1.66 | 1.48-1.87 | <0.001 |
| Year 14 | 1505 | 296 (19.67) | 9216 | 907 (9.84) | 257.19 | 127.31 | 1.67 | 1.46-1.92 | <0.001 |
| Year 15 | 823 | 205 (24.91) | 5156 | 628 (12.18) | 364.33 | 177.19 | 1.75 | 1.49-2.06 | <0.001 |

*White race, female sex, non-smokers, patients without gallstone disease, and control patients without pancreatic cancer were taken as a reference category.

**Table S3.** Patient characteristics by diabetes status

|  | **New-onset Diabetes** | **Controls** | **P-value** | **All study patients*** |
| --- | --- | --- | --- | --- |
| N (%) | 1199477 (100) | 4115782 (100) |  | 5315259 |
| Age in years (Median, interquartile range) | 62 (55-72) | 64 (53-74) | <0.001 | 63 (53-74) |
|  |  |  |  |  |
| Sex |  |  | <0.001 |  |
| Male | 1138690 (94.9) | 3803162 (92.4) |  | 4941853 (93.0) |
| Female | 60787 (5.1) | 312619 (7.6) |  | 373406 (6.3) |
|  |  |  |  |  |
| Race |  |  | <0.001 |  |
| White | 898413 (75.8) | 3231156 (83.9) |  | 4129569 (82.0) |
| Black | 227554 (19.2) | 481894 (12.5) |  | 709448 (14.1) |
| Other | 59053 (5.0) | 137855 (3.6) |  | 196908 (3.9) |
|  |  |  |  |  |
| Smoking |  |  | <0.001 |  |
| Current | 515643 (43.3) | 1129640 (35.5) |  | 1645283 (37.6) |
| Past | 227125 (19.1) | 793241 (24.9) |  | 1020366 (23.3) |
|  |  |  |  |  |
| Alcohol | 316251 (26.4) | 508576 (12.4) | <0.001 | 824827 (15.5) |
|  |  |  |  |  |
| Acute Pancreatitis | 21267 (1.8) | 8867 (0.2) | <0.001 | 30134 (0.6) |
| Chronic Pancreatitis | 14159 (1.2) | 8952 (0.2) | <0.001 | 23111 (0.4) |
| History of gallstones | 69639 (5.8) | 60560 (1.5) | <0.001 | 130199 (2.5) |
| Pancreatic cancer | 7161 (0.6) | 5160 (0.1) | <0.001 | 12321 (0.3) |

Age-age at the time of entry into the study, *race missing in ~4% of all pts included in the study

**Table S4.** Annual incidence and risk (hazard ratio) of pancreatic cancer by new-onset diabetes status

|  | **New-onset Diabetes (NODM)** | | **Controls** | | **Incidence per 1000 patient years** | | **Risk** | | |
| --- | --- | --- | --- | --- | --- | --- | --- | --- | --- |
|  | Patients at risk (n) | Patients diagnosed with PDAC | Patients at risk (n) | Patients diagnosed with PDAC | NODM | Controls | Adjusted Hazard Ratio | 95% Confidence Interval | P value |
| Year 1 | 1199477 | 2814 (0.23) | 4115782 | 777 (0.02) | 2.50 | 0.20 | 9.07 | 8.33-9.87 | <0.001 |
| Year 2 | 1064032 | 948 (0.09) | 3782494 | 687 (0.02) | 0.94 | 0.19 | 3.40 | 3.06-3.77 | <0.001 |
| Year 3 | 954287 | 684 (0.07) | 3446213 | 561 (0.02) | 0.76 | 0.17 | 3.09 | 2.74-3.48 | <0.001 |
| Year 4 | 847602 | 561 (0.07) | 3113712 | 520 (0.02) | 0.70 | 0.18 | 2.71 | 2.38-3.08 | <0.001 |
| Year 5 | 744981 | 513 (0.07) | 2807977 | 482 (0.02) | 0.74 | 0.18 | 2.76 | 2.42-3.15 | <0.001 |
| Year 6 | 642208 | 426 (0.07) | 2548966 | 428 (0.02) | 0.72 | 0.18 | 2.72 | 2.36-3.14 | <0.001 |
| Year 7 | 542618 | 349 (0.06) | 2304779 | 401 (0.02) | 0.71 | 0.18 | 2.41 | 2.06-2.82 | <0.001 |
| Year 8 | 444628 | 284 (0.06) | 2060032 | 334 (0.02) | 0.71 | 0.17 | 2.64 | 2.22-3.13 | <0.001 |
| Year 9 | 357753 | 212 (0.06) | 1825212 | 306 (0.02) | 0.67 | 0.18 | 2.40 | 1.99-2.90 | <0.001 |
| Year 10 | 271960 | 190 (0.07) | 1580666 | 238 (0.02) | 0.82 | 0.16 | 3.33 | 2.71-4.09 | <0.001 |
| Year 11 | 192038 | 99 (0.05) | 1311330 | 192 (0.01) | 0.63 | 0.16 | 2.46 | 1.89-3.19 | <0.001 |
| Year 12 | 120930 | 71 (0.06) | 1025560 | 136 (0.01) | 0.81 | 0.15 | 3.04 | 2.23-4.16 | <0.001 |
| Year 13 | 56682 | 10 (0.02) | 739542 | 98 (0.01) | 0.30 | 0.17 | 0.79 | 0.40-1.58 | 0.513 |
| Year 14 | 11593 | 0 | 377294 | 0 | - | - | - | - | - |

* versus controls (remaining patients in the database without NODM), adjusted for age (age at the time of entry into the study), gender, race, smoking, alcohol, gallstones, and diabetes. PDAC- pancreatic cancer. NODM- New-onset diabetes mellitus

**Table S5.** Predictors for pancreatic cancer diagnosis following New-Onset Diabetes Mellitus

|  | Odds ratio | 95% Confidence Interval | P value |
| --- | --- | --- | --- |
| African American Race | 1.07 | 1.00-1.14 | 0.047 |
| Other Race | 0.95 | 0.84-1.06 | 0.339 |
| Male sex | 1.37 | 1.19-1.58 | <0.001 |
| Age | 1.03 | 1.03-1.04 | <0.001 |
| Current smoker | 1.24 | 1.17-1.31 | <0.001 |
| Past smoker | 0.97 | 0.91-1.04 | 0.414 |
| Gallstone disease | 1.87 | 1.74-2.01 | <0.001 |
| AP | 2.89 | 2.62-3.20 | <0.001 |
| CP | 6.65 | 6.03-7.33 | <0.001 |

*White race, female sex, non-smokers, patients without gallstone disease and chronic pancreatitis and acute pancreatitis were taken as a reference category

**Table S6.** Summary of previous studies evaluating the risk of PDAC in NODM patients

| **Authors** | **Study period** | **Database** | **NODM** | **PDAC within 3 years** | |
| --- | --- | --- | --- | --- | --- |
|  |  |  | **n** | **n** | **%** |
| Brodovicz et al. | 2003-2009 | Medicines and Healthcare Product Regulatory Agency, UK | 146692 | 302 | 0.21% |
| Boursi et al. | 1995-2013 | The Health Improvement Network, UK | 109,385 | 390 | 0.36% |
| Jensen et al. | 1998-2018 | Danish National Patient Registry, nationwide cohort, Denmark | 353,970 | 2105 | 0.59% |
| White et al. | 2007-2015 | administrative insurance claims data from the OptumLabs, USA | 640,421 | 579 | 0.09% |
| Chari et al. | 1950-1994 | Rochester Epidemiology Project, Olmsted County, USA | 2,122 | 18 | 0.85% |
| Setiawan et al | 1993-2014 | Multiethnic Cohort Study, Hawaii and California, USA | 15,833 | 68 | 0.43% |
| Sharma et al. | 2000-2015 | Rochester Epidemiology Project, Olmstead County USA | 1,561 | 16 | 1.02% |
| Munigala et al | 1999-2007 | VA database, USA | 73,811 | 183 | 0.25% |
| Chen et al. | 2010-2014 | Kaiser Permanente Southern California, USA | 18,541 | 99 | 0.53% |
| Illes et al.* | 2012-2014 | Diabetic clinic, University of Szeged, Hungary | 115 | 3 | 2.61% |
| Lu et al | 1996-2010 | The Health Improvement Network, UK | 44,373 | 175 | 0.39% |
| Tseng | 1996-2005 | National Health Insurance, Taiwan | 19,793 | 19 | 0.10% |
|  |  | **Total** | **1,432,871** | **3957** | **0.28%** |

*New-onset diabetic patients enrolled from an outpatient diabetic clinic.

**Table S7.** Incidence of Pancreatic ductal adenocarcinoma (PDAC) in patients with New Onset Diabetes Mellitus (NODM) diagnosed based on glycemic parameters, ICD9 codes or both

| **Group** | **NODM diagnosis based on** | | **Patients (n)** | **PDAC (n (%))** |
| --- | --- | --- | --- | --- |
|  | **Glycemic parameters** | **ICD 9 Codes** |  |  |
| I | Positive | Positive | 465,408 | 2891 (0.62%) |
| II | Positive | Negative | 734,069 | 4270 (0.58%) |
| III | Negative | Negative | 4,115,782 | 5160 (0.13%) |

**
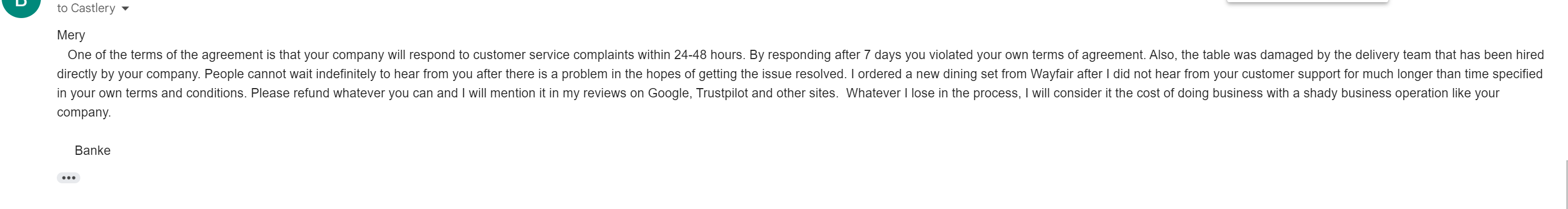

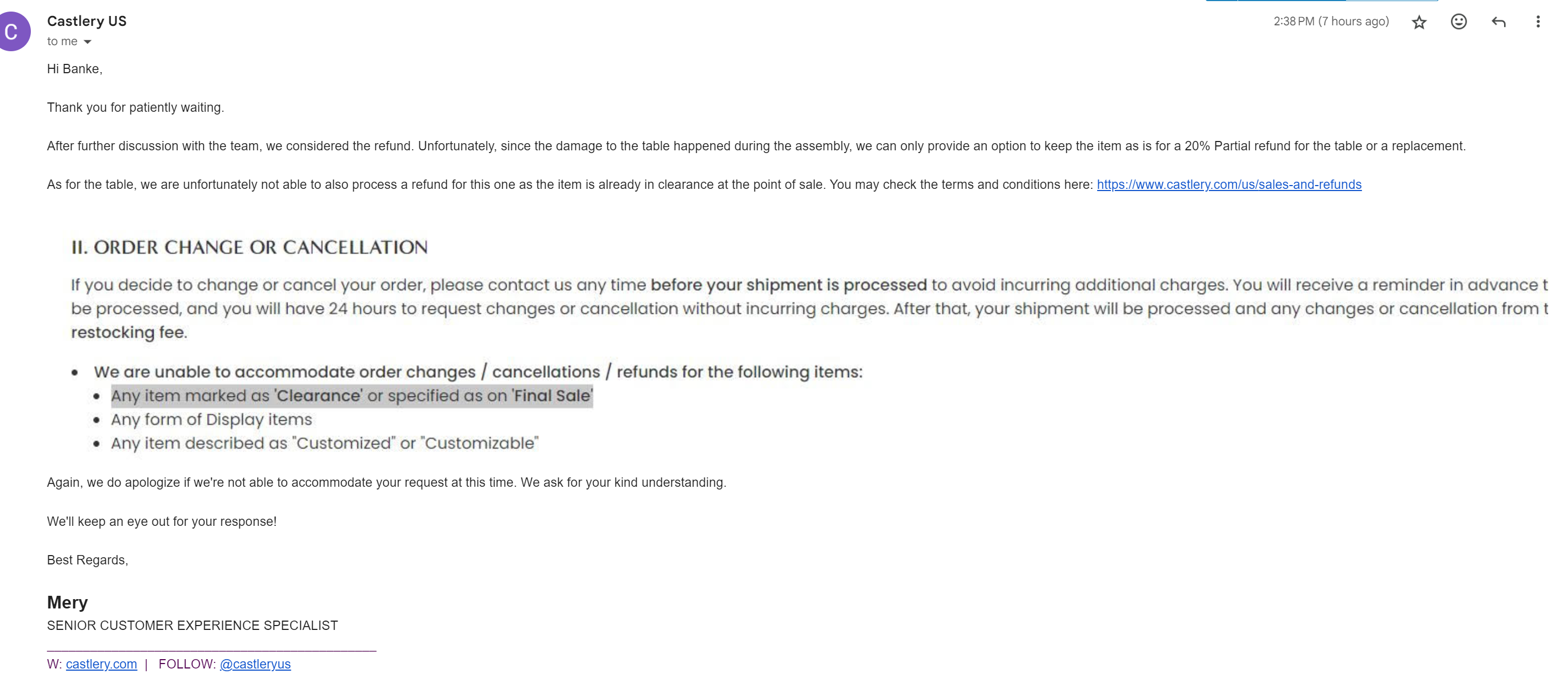
Note:** 1,045,654 patients met the inclusion criteria (based on ICD codes and/or glycemic parameters) but did not have availability of normal glycemic results in the two previous years to be able to qualify for inclusion as ‘New Onset Diabetes Mellitus’. 4337 (0.41%) of these patients were diagnosed with pancreatic cancer in the following years during the study duration. These patients were excluded and were not part of either the NODM or the Control group.
