# Supplementary material for: Assessing NODM Patients for Early PDAC Diagnosis: Incidence of NODM Before PDAC Diagnosis and Subsequent PDAC Risk
Source: Cancer Med. 2025 May 12;14(9):e70878. doi: 10.1002/cam4.70878 (PMC12066942; doi:10.1002/cam4.70878)
Supplement: Supplementary file 2 — Data S1: [file CAM4-14-e70878-s002.docx]

**Study part I: Case-control study evaluating the annual incidence of NODM prior to PDAC diagnosis.**

Cohort Selection

We identified patients with PDAC who had ≥10-year follow up in the VA system prior to PDAC diagnosis (n= 8,198). Age- and sex- matched controls (1:5 matching) with follow-up in the VA system for preceding ≥10-years (n=40,992) were identified. The index date for the control subjects was the date of PDAC diagnosis of the patient with whom they were matched. The exclusion criteria for this case-control study are illustrated in Figure 1a.

Statistical analysis

Patient characteristics of both groups were assessed and compared using frequencies (n (%)) and age was compared using median ±interquartile range (IQR). The annual incidence of NODM prior to PDAC diagnosis in patients with PDAC and in controls in years prior to the index date was calculated. Cox-proportional hazards model analysis was performed and hazard ratios (HR) for NODM, both unadjusted and adjusted (aHR) for demographic and other covariables, were calculated for each year and for the entire Duration of Excess Risk (DER).

**Study part II: Evaluating the annual incidence of PDAC diagnosis in the years following NODM.**

Exclusion criteria

We excluded patients with 1) pre-existing DM (using the diagnosis of DM from Table S1) in the preceding 3 years (n=1,867,592), 2) PDAC (n=7,658) diagnosed within three years prior to entry into the study cohort and 3) veterans with <3 years of follow-up in the system (n=1,673,078). Patients <40 years of age (n=1,889,257) were not considered as their risk of PDAC is extremely low.^19^ Final study cohort comprised 6,360,913 veterans (Figure 1b).

Retrospective cohort assembly

Based on the NODM status, the selected veterans were classified into NODM group and control group (remaining patients without NODM). These patients were followed in time to evaluate for annual incidence of PDAC. For patients in the NODM group, date of first glycemic parameter was designated as the time of study entry (T0) and for controls the first outpatient or inpatient encounter after 3-year washout period was designated as the time of study entry.

Follow-up for those who developed PDAC ended on the date of first diagnosis of PDAC, and for those who did not develop PDAC it was censored at the time of loss to follow-up (last visit recorded in the VA system during the study period), death, or end of the study period.

Statistical analysis

For the longitudinal analysis, patient characteristics for NODM vs control groups were assessed using frequencies (n (%)) and age was compared using median ± IQR. The proportion of patients who were subsequently diagnosed to have PDAC was calculated, and incidence rates (per 1000 person-years) were estimated. Cause-specific hazard model was fitted using Cox-regression model (competing events were treated as censored observations) and hazard ratios ((HR) unadjusted, and after adjusting (aHR) for demographic and other covariates) with 95% confidence intervals (CIs) were calculated for the PDAC risk for each year and for the entire study duration. We also evaluated the predictors for PDAC among NODM patients. Cumulative incidence curves of PDAC were generated for NODM patients and controls.

All analyses were conducted using SAS version 9.3 (SAS Inc, Cary, NC). Significance tests were performed by using a 2-tailed hypothesis and the level of significance (α) was set to 0.05. This study was approved by the Saint Louis Veterans Affairs Medical Center.
